# Potential Using of Fat-derived Stromal Cells in the Treatment of Active Disease, and also, in Both Pre- and Post-Periods in COVID-19

**DOI:** 10.14336/AD.2020.0621

**Published:** 2020-07-23

**Authors:** Hasim Eray Copcu

**Affiliations:** Mest Health Services, Aesthetic and Plastic Surgery, Izmir, Turkey

**Keywords:** COVID 19, stromal cells, stem cell, ADSC, adipose tissue

## Abstract

The whole world is fighting with the COVID-19 pandemic, which traps people home, causing high business and economic losses, and above all, leads to very serious deaths. The lack of a valid, accepted treatment protocol and vaccine that leads to continued treatment searches. Leng et al published their article in the Aging and Disease journal, which demonstrates that mesenchymal stem cells (MSCs) can be used for COVID-19 treatment. Adipose tissue is one of the most important MSCs sources in the body, and adipose derived stromal cells (ADSCs) from adipose tissue are also one of the most valuable components of stromal vascular fraction (SVF). Finally, Gentile and Sterodimas, have also published their article for the potential use of SVF in COVID-19 treatment in Aging and Disease journal. Their publication has been a guide in many ways. Adipose tissue-derived stromal cells have three main features: Immunomodulatory, anti-inflammatory and regenerative. Immunomodulator effects are used as a preventive in patients prone to disease; its anti-inflammatory effects may allow them to be used as a therapeutic during active disease period and finally regenerative effects to repair post-disease sequale. Those cells can be obtained not only enzymatically, but also mechanically with very benefits. They can be delivered not only systemically through the IV route but also to the target organ with a carrier. While suggesting any adipose tissue-derived treatment method possibility, the relation of adipose tissue COVID-19 should not be ignored. Because, COVID-19 shows its effect through ACE-2 and adipose tissue is very rich and important tissue in terms of ACE-2.

According to the latest data, the COVID-19 pandemic, which affects 210 countries in the world, has infected approximately 8.6 million people as of June 17 and caused nearly 457.000 registered deaths (www.worldometers.info/coronavirus/?utm_campaign=homeAdvegas1?). This situation changed the balance of the whole world; in addition to three hundred thousands of deaths, it created a global economic loss of $ 3.5 trillion and fear of infection that affects all people (www.statista.com/topics/6139/covid-19-impact-on-the-global-economy/). This is the biggest crisis that human beings experienced in the 21^st^ century. Big crises, such as war or this kind of pandemics, can provide new treatment hopes or advance existing treatment methods. One of the best examples of this idea is that Morrestin experiences which promoting the healing in WW1, by transferring the fat tissue particles into the wounds of the injured soldiers [1]. Thus, it actually used the regenerative effect of fat tissue. Later, although in 1986 Jarrell [2] presented the effect of microvascular endothelial cells from adipose tissue in his study, this issue was mainly popularized by Zuk *et al.* in 2001 [3]. They presented that fat tissue is a very important and the largest mesenchymal stem/stromal cell source of body.

Similarly, stem cell applications, which are accepted as the treatment of the near future, have become increasingly important in the treatment of COVID-19. This topic was discussed in the medical literature started with the work of Leng et al in Aging & Disease journal [4]. They published the results of seven patients suffering from COVID-19 infection with varying clinical situations and treated with clinical grade human MSCs. In the same journal, first, Shetty's editorial response was published [5]. Author criticized the paper of Leng *et al* and concluded that intravenous infusion of MSCs is a safe and efficient approach for treating patients with COVID-19 pneumonia, including in elderly patients displaying severe pneumonia. Immediately after this publication, 2 other editorials were also published in Aging & Disease journal. The first one was the commentary of Ozturk *et al.* They focused the acute respiratory distress syndrome (ARDS) caused by COVID-19 [6]. They stated that therapeutic role of MSCs in ARDS mostly originates from animal model studies suggesting a repair process through modulation of immune system cells and inflammatory/ anti-inflammatory bioactive molecules [6]. Similarly, in his paper, Chen examined the similarities between epidemic Influenza and COVID-19 clinics, and concluded that both H7N9 infected patients and COVID-19 infected patients share similar symptoms including cough, fever, shortness of breath, sputum, and dyspnea accompanied by ARDS or later pulmonary fibrosis, some patients with severe symptoms with ARDS might benefit from novel methods including MSC-based therapy [7]. Also Ozturk *et al.* presented clinical trials recorded in www.clinicaltrials.gov databases about stem cells and Covid-19 infection [6]. There were 9 different studies by the 27^th^ of March 2020 in their study, but as of June 19, 2020, this number was 49. In other words, the number of registered studies increased by approximately 500% over a period of 1 month. This is an indication that stem cell therapy will find a good place in the future (www.clinicaltrials.gov). Ozturk *et al.* concluded that good outcomes from MSCs in COVID-19 infection due to their close intersections regarding pathogenesis of the disease and mechanism of action of MSCs [6]. Finally, an editorial was published by Gentile and Sterodimas in same journal. They presented for the first time that ASC and SVF can be used in the treatment of COVID-19 from a different perspective [9]. They suggested the possibility to use autologous or allogenic ASCs intravenously or directly through a ventilation mask (aeresol) [8] .With this article, we would like to look at the subject from a different point of view, and for different purposes, stromal cells originating from adipose tissue can be used and present possible advantages and disadvantages.

## Stem cells/Stromal cells?

There are two types of cells in all organs that make up our body—parenchymal cells responsible for the function of the organ and stromal cells that support them. Stromal cells provide tissue repair and renewal following stress, injury, illness, or aging, that is, from both intrinsic and extrinsic causes [9]. Mesenchymal stromal cells are defined by the International Society of Cellular Therapy (ISCT) by their (1) ability to adhere to plastic. from which they can be expanded in culture in serum containing medium, (2) cell surface immunophenotype (positive for CD13, CD73, CD90, CD105 and HLA-Class I, and negative for CD45, CD11b, CD14, CD31, CD34), and (3) ability to differentiate into mesenchymal-derived tissue (adipogenesis, chondrogenesis and osteogenesis [10]. MSCs are not a pure population of stem cells; the ISCT criteria actually describe MSCs as a heterogeneous, nonclonal mix of multipotent stem cells, committed progenitors, and differentiated cells. This fact prompted a change in nomenclature from ‘‘mesenchymal stem cell’’ to ‘‘mesenchymal stromal cell’’ to better reflect their cellular heterogeneity, although the terms are still used interchangeably [11].

MSCs attract particular attention due to their broad pharmacological effects, including anti-inflammatory, immunomodulatory, regenerative, pro-angiogenic and anti-fibrotic properties [12]. MSCs are multipotent cells that can differentiate into a variety of cell types, including osteoblasts, chondrocytes, myocytes and adipocytes, depending on environmental cues. MSC can be identified in association with the vasculature of virtually every organ and are prevalent in highly vascularized adipose tissue [13].

## MSC vs. ADSC

Adipose tissue is a very important mesenchymal stromal cell source, and stromal cells derived from adipose tissue are called ADSC. ADSCs are unique from bone marrow-derived MSCs (BM-MSCs) by the expression of the fatty acid translocase marker CD36 and the lack of expression of cell adhesion marker CD106 [10]. The adipose secretome generates adipokines and cytokines with both local and systemic effects- abilities that extend to isolated ADSCs. ADSC conditioned media, a direct product of the ADSC secretome, has been demonstrated in several studies to exert there endocrine and paracrine effects. Compared with BM-MSCs, ASCs have a higher differentiation capacity, proliferation rate, and regenerative potential [14]. Adipose derived stromal cells have high immunomodulating capacity and can ameliorate lung injury by secreting several factors with paracrine effects of several growth factors, such as hepatocyte growth factor (HGF), vascular endothelial growth factor (VEGF), and fibroblast growth factor-2 (FGF-2) [15]. It has been proven that ADSCs have beneficial effects in animal models of pulmonary diseases such as COPD, ARDS, bronchiolitis obliterans, idiopathic pulmonary fibrosis and emphysema [14]. These paracrine effects promote the proliferation of alveolar epithelial cells and angiogenesis and inhibit cell apoptosis [15]. ADCs will have the greatest support in the treatment of COVID 19, especially in terms of lung involvement, in terms of type 2 alveolar cell regeneration. Fukui et al, showed that that ADSCs differentiate into type 2 alveolar epithelial cells [15]. Additionally, AD-MSCs seem to have greater anti-inflammatory potential than BM-MSCs, since they secrete higher levels of bioactive mediators, and have been shown to attenuate lung inflammation in different models of lung injury [16]. In experimental pulmonary system models, the causes of unsuccessful MSC treatment have been reported as insufficient cell number, condition of cells, cell types, frequency and timing of administration and insufficient doses of administration [15,17]. These problems can be solved with stromal cells from adipose tissue. Because a large number of cells can be obtained in high quality and not only as MSC, but also as a cocktail with other stromal cells.

## ADSC vs SVF

Adipose tissue is the largest reservoir in the body for MSCs and enables quality stromal cell production. Obtaining ADSC is much easier and cheaper than any other MSC. It does not cause any problems for both the machine and the experienced team. A ready-to-use, fresh, autologous stromal cell is obtained at the bedside in a very short time. This feature is very important in COVID-19 treatment because clinical trials almost universally use freshly thawed MSC stocks; however, cryo-preservation appears to impair the immunosuppressive properties of MSCs and shorten their persistence *in vivo* [9]. Stromal cells from adipose tissue can be obtained by two methods: enzymatic and mechanical. Classically, in the method by which both ADSCs and other stromal cells are obtained using the enzyme, the end product is called SVF. When stromal cells are obtained from adipose tissue using enzymes, expensive machines are required and cGMP and cGLP standards are mandatory. However, obtaining stromal cells mechanically recently has been quite popular and has many advantages: Easier and simpler, cheaper but above all cell-cell connections, ECM and total stromal cells are preserved [18]. There is no description in the literature about the end product containing stromal cells obtained mechanically; we suggest that the final product containing ADSC and stromal cells obtained by mechanical method is called Total Stromal (TOST) cells, because SVF is a definition for enzymatic methods and all stromal cells are protected in mechanical methods. What makes SVF and TOST, different from MSC or ADSC is a tissue cocktail containing many stromal cells. These cells pericytes, vascular progenitors, and multipotent mesenchymal stem cells [10]. Although SVF and TOST has many promising potential uses, its most important feature is that it is an immunomodulatory. The ability of these cells to suppress an immune response has obvious indications for autoimmune disorders, these anti-inflammatory effects have been exploited in studies of several pathologic processes, such as hemorrhagic stroke, reactive airway disease, multiple sclerosis, and rheumatoid arthritis [19].

## Mechanism of Action of Adipose Derived Stromal Cells

The immunomodulatory effects of stromal cells have been primarily characterized as immunosuppressive, anti-inflammatory and tolerogenic. These 3 main features allow the use of stromal cells for 3 different periods: 1. Pre-infection period: Prevention, 2. Infection Period: Active disease treatment 3. Post-infection period: repair sequels and regeneration of organs affected. Basically, these effects are performed as an immunomodulatory as propagated by resident cells such as macrophages, T cells, and stromal cells.

## PRE-INFECTION PEROD: PREVENTION

The best way to prevent COVID-19 infection is undoubtedly the vaccine. Unfortunately, however, still no vaccine has been found. A balance between pro- and anti-inflammatory mechanisms is critical in maintaining lung tissue homeostasis, therefore preventive topical/systemic stromal cell applications might be beneficial. A recent report from Wuhan suggests that SARS-CoV-2 induces a dysregulated immune response in severely ill COVID-19 subjects, characterized by reduced numbers of circulating memory T lymphocytes, as well as reduced helper / suppressor T cell and T Reg subtypes. It is tempting to speculate whether already dysfunctional immune responses in subjects with obesity may accentuate this SARS-CoV-2 effect on T cell function. [20] ADSCs further promote immune tolerance by secreting TGF-β1 and galectin-1 and galectin-3, paracrine factors that induce differentiation of T-helper cells into T-suppressor cells [21]. The ability of ADSCs to regulate and influence the frequency and activity of T cells, B cells, and macrophages has been documented in numerous in vitro coculture systems and in vivo models. ASCs, similar to BM-MSCs, inhibited allogeneic lymphocyte proliferation in mixed lymphocyte cultures through cell-cell contact; one of the possible mechanisms mediating the immunosuppressive effect of ASCs is via stimulating the production of functional de novo regulatory T cells [15]. Immunoregulation of ASCs is based on direct cell-cell contact with immune cells associated with paracrine secretion of soluble mediators, cytokines, and growth factors [15]. Direct cell-cell transmission of mitochondria from stromal cells to respiratory epithelial and immune cells has also been described [22]. MSC administration n has been demonstrated to protect or even reduce morphological and functional abnormalities in the lung and infection is the most common cause of ARDS, and although stromal cells themselves lack phagocytic activity, they can stimulate phagocytosis by host immune cells and production of antimicrobial peptides and also they can be used prophylactic to improve immune system to the infections [23]. Hong et al. reported that ADSCs pretreated with the peroxisome proliferator-activated receptor-γ agonist, pioglitazone, have more potent therapeutic effects than non-pre-treated ADSCs for the repair of alveolar destruction in emphysema mouse models [15].

### Infection period: active infection treatment

Neighboring cells from oxidative stress and can support the viability and activity of neutrophils. Thus, ADSCs may help promote early microbial clearance while protecting native tissues. Interestingly, exogenously delivered ADSCs are known to express MCP-1, suggesting a potential mechanism for improved healing by supporting monocyte recruitment to the wound bed [24]. Adipose derived stromal cell therapy can prevent storm release of cytokines by the immune system and promote endogenous repair by reparative properties of the stem cells. Huang and colleagues demonstrated that, after entering the cells, the virus could stimulate a terrible cytokine storm in the lung, increasing the levels of interleukin (IL)-2, IL-6, IL-7, granulocyte colony-stimulating factor (GSCF), interferon *γ*-induced protein 10 (IP10), monocyte chemoattractant protein-1 (MCP1), macrophage inflammatory protein (MIP1A) and tumor necrosis factor-alpha (TNF-α) [5]. The therapeutic strategies are only supportive and oxygen therapy represents the primary treatment intervention for patients with severe pneumonia. Mechanical ventilation is necessary in cases of respiratory failure. The key to save the patients with severe COVID-19 pneumonia may be, in addition to inhibiting viral replication, preventing and reversing the cytokine storm. Here, secretome spread into the tissues and it provided immune modulation, resolution of inflammation, restoration of capillary barrier function and enhanced bacterial clearance [25]. A growing literature demonstrates that the pattern of anti-inflammatory mediators released is specific for the inflammatory lung environment encountered and is mediated through differential activation of damage and pathogen-associated molecular pathogen receptors expressed on MSC cell surfaces [22]. MSCs also play a role in the control of tissue inflammation. In response to inflammatory factors such as Interferon (IFNγ) and Tumour Necrosis Factor (TNFα) secreted by activated immune cells and tissue cells, MSCs adopt an immunoregulatory phenotype. They elevate the expression of anti-inflammatory factors including programmed death ligand 1 and prostaglandin E2 and inhibit immune cell activity and proliferation through metabolic regulation, such as via indolamine 2,3-dioxygenase dependent catabolism of tryptophan [26]. Administration of MSCs either intratracheally or intravenously has been demonstrated to mitigate inflammation by reducing levels of several inflammatory mediators, including in- terleukin (IL)-1-α, IL-1β, IL-6, IL-8, IFN-γ, macro- phage inflammatory protein (MIP)-1, MIP-2, and tumor necrosis factor (TNF)-α, while also increasing levels of anti-inflammatory and pro-resolution factors, such as IL-1 receptor antagonist (IL-1RN), IL-10, prostaglandin E2 (PGE2), lipoxin A4 (LXA4), and TNF-inducible gene (TSG)-6 [23].

### Post-infection period: regeneration

The regenerative approaches have included extrinsic cell therapy such as the infusion of exogenous stem cells to repair the damaged structure of the respiratory system and intrinsic cell therapy such as the administration of small molecules to stimulate the endogenous lung stem/ progenitor cells for regeneration and replacement of the damaged structures [17]. Adipose derived stromal cells , using their immunomodulatory properties and their differentiation ability, can prevent lung tissue death by counteracting the cytokine storm and regeneration and reconstruction of damaged tissues . Notably, it has been proved that MSC-released cytokines can potently inhibit neutrophil intravasation and enhance the differentiation of macrophages. Moreover these, MSC-released EVs can deliver microRNA, mRNA, DNA, proteins, and metabolites into host cells in specific injuries of the lung to promote lung repair as well as regeneration and restore lung function [27].

### Methods of applying stromal cell therapy

One of the key benefits of MSC-based therapies is their ability to preferentially home damaged tissues. It is worth elaborating on what exactly we mean by homing, as the term is often used vaguely in the literature. Following the recommendations of previous reviews, we take homing to encompass both non-systemic and systemic homing. In non-systemic homing, MSCs are transplanted locally at the target tissue and are then guided to the site of injury via a chemokine gradient. In systemic homing, MSCs are administered or endogenously recruited into the bloodstream and then must undergo a multi- step process to exit circulation and migrate to the injury site [26]. Two principle delivery routes were systemic (intra-venous route) delivery and local (intra-tracheal /intra-bronchial routes). The most criticized aspect of MSCs in therapeutic use as IV was lung trapping. This is a negative situation for the delivery of cells to the targeted tissue. However, this may be an advantage in coivd19 treatment. Because in Covid19 infection, the life-threatening condition is that the virus affects the lungs and creates an ARDS-like clinic. The most used route of administration for COPD therapy is IV infusion. Adipose-derived stromal cells can also be applied directly to the lung tissue. For this, intra-tracheal / intra-bronchial applications can be preferred. In this case, fibrin such as biocompatible matrix can be preferred [28]. This approach can be preferred especially in the production of stromal cells mechanically. Rubin et al, presented that adipose derived stromal cells can be administered and immobilized in a fibrin matrix through an aerosol delivery system while preserving the ability to grow and differentiate [28]. This may be a suitable alternative for direct administration of adipose-derived stromal cells to the lung either by enzyme or mechanically. The heterogeneity and rich stem/progenitor cell content of freshly isolated SVF cells, and their ability to differentiate along adipogenic and endothelial lineages after Tisseel delivery, suggest that they may participate in immunomodulation, regeneration, angiogenesis and anti-inflammatory response when applied in pulmonary system in Covid19 patients.

### Action Mechanism of COVID-19 and Adipose Tissue / Stromal Cells

The SARS-CoV-2 virus uses as a cellular entry the angiotensin-converting enzyme II (ACE2) receptor , which is widely distributed on the alveolar type II cells and capillary endothelium of the lungs, as well as in many other organs, including the cardiovascular, liver, kidney and gastrointestinal tract [25]. For this reason, ACE2-positive cells are infected by this virus. Another study has shown that the cellular protease TMRRSS2 is also required to allow the entry of coronavirus into host cells [29]. It is conceivable that the ACE2 receptor is widely distributed on the surface of human cells, especially alveolar type 2 (AT2) and capillary epithelium, and AT2 cells largely express TMPRSS2 [29]. The current hallmark of SARS-CoV-2 pathogenesis is the cytokine storm in the lung. Virally-triggered acute cytokine release of GSCF, IP10, MCP1, MIP1A, IL-2, IL-6, IL-7, and TNF results in pulmonary edema, dysfunction of air-exchange, acute respiratory distress syndrome (ARDS), and acute cardiac injury, and leading to death [30] RAS components were identified in the late 1980s in the adipose tissue and were found to be present both in human and rodent adipose tissue as well as cultured adipocytes [31]. Specifically, human WAT expresses Agt, renin, ACE, ACE2, AT1R, AT2R, (P)RR, and Mas receptor [32]. The main precursor of RAS, Agt is expressed in WAT in both animal and human models [33]. Currently, there is no evidence for direct SARS-CoV-2 infection of AT, although ACE2 receptor expression represents a basis for viral tropism in several cells within this tissue including adipocytes, smooth muscle cells and endothelial cells [20]. Moreover, many AT-resident cells are proven targets for multiple viruses including adipocytes (H1N1, Type A influenza and adenovirus 36, adipostromal cells (Adenovirus 36, CMV), endothelial cells (SARS-CoV), macrophages (influenza A, SARS-CoV, adenovirus 36, HIV) and lymphocytes (SARS-CoV, HIV) [21]. Experimental studies have revealed that RAS expressed in the adipose tissue is implicated in the regulation of the adipocyte formation and supported a role for Ang II as a negative regulator of adipogenesis [34]. It is reported that adipose tissues could be infected by some virus, like H5N1, HIV. Furthermore, obesity could influence the mortality and transmission of influenza virus. To date, no research has demonstrated that SARS-CoV-2 could infect adipose tissue. Moreover, there is still no evidence to show that obesity is associated with COVID-19. The authors found the expression of ACE2 in gallbladder and adipose tissue (including subcutaneous and visceral) is significantly higher than that in lung [35]. Stromal cells are generally resistant to viral infection compared to their differentiated progeny [30]. The reason that immunotherapy can be a safe method in Covid19 is the structure of immune cells. Bone marrow, lymph nodes, thymus, spleen, and immune cells, such as T and B lymphocytes and macrophages are always negative for ACE2 [30].

ACE and RAS system are of great importance in the formation of hypertrophic adipocytes. The classical RAS has been found to be over-activated during the AT enlargement, thus elevated generation of angiotensin II (Ang II) may contribute to the obesity pathogenesis [36]. Therefore, adipocytes of normal size, not hypertrophic ones, should be preferred in order not to experience a possible problem for the fat graft surgery in aesthetic plastic surgery. This can only be achieved by removing hypertrophic fats from the environment with sharp blade systems. In other words, it is inevitable to define "organic" fat grafting with a popular term.

### Conclusion

Adipose-derived mesenchymal stromal cells can potentially be used not only for the treatment of COVID-19 active disease, but also for pre-disease protection or for regeneration of the affected organ after disease. SVF or TOST is a perfect combination of mesenchymal stromal cells. The use of this tissue cocktail, rather than mesenchymal stem cells alone, can have many advantages both for the practitioner and the patient. The application can be done on an IV route or an organ directly affected by a suitable carrier. In the light of current literature data, there is no negativity regarding the use of stromal cells obtained from adipose tissue in COVID 19. However, as adipocyte hypertrophy is associated with ACE 2, the application of hypertrophic adipocytes as grafts can be a potential problem. Normal adipocytes, not hypertrophic ones, should be preferred in order not to experience possible problems regarding the use of fat tissue itself in terms of plastic surgery. Using sharp blade systems, without blunt pressure, without killing adipose tissue, hypertrophic adipocytes are cut away and normal adipocytes can be obtained. This is the “new normal” that COVID-19 causes for the most applied fat grafts in aesthetic plastic surgery.
